# Impact of thyroid-stimulating hormone levels after controlled ovarian hyperstimulation on *in vitro* fertilization/intracytoplasmic sperm injection outcomes in women with fresh embryo transfer: a prospective cohort study

**DOI:** 10.3389/fendo.2023.1159991

**Published:** 2023-08-29

**Authors:** Ning Huang, Lixue Chen, Ying Lian, Hongbin Chi, Jie Qiao

**Affiliations:** ^1^ Center for Reproductive Medicine, Department of Obstetrics and Gynecology, Peking University Third Hospital, Beijing, China; ^2^ National Clinical Research Center for Obstetrics and Gynecology, Peking University Third Hospital, Beijing, China; ^3^ Key Laboratory of Assisted Reproduction, Peking University, Ministry of Education, Beijing, China; ^4^ Beijing Key Laboratory of Reproductive Endocrinology and Assisted Reproductive Technology, Beijing, China; ^5^ Beijing Advanced Innovation Center for Genomics, Peking University, Beijing, China; ^6^ Peking-Tsinghua Center for Life Sciences, Peking University, Beijing, China

**Keywords:** *in vitro* fertilization, intracytoplasmic sperm injection, controlled ovarian hyperstimulation, clinical pregnancy, miscarriage, live birth, preterm delivery

## Abstract

**Objective:**

Maternal hypothyroidism before and during pregnancy is associated with an increased risk of adverse pregnancy outcomes; many studies have evidenced that controlled ovarian hyperstimulation (COH) triggers a significant increase in the levels of TSH; however, no large-scale prospective studies have evaluated the impact of TSH levels after COH on assisted reproductive technology outcomes. The aim of this prospective study was to investigate whether *in vitro* fertilization/intracytoplasmic sperm injection (IVF/ICSI) outcomes are affected by TSH levels after COH in women with fresh embryo transfer (ET).

**Methods:**

A total of 664 patients who underwent IVF/ICSI treatment and received fresh ET at the Peking University Third Hospital were included in this study. The rates of clinical pregnancy, miscarriage, live birth, and preterm delivery were analyzed.

**Results:**

The patients were categorized into two groups based on serum TSH levels after COH (0.55 mIU/L < TSH < 2.5 mIU/L: n= 449, 2.5 mIU/L ≤ TSH ≤ 4.78 mIU/L: n= 215). There were no significant differences in the rates of clinical pregnancy, miscarriage, and live birth between the two groups, even after adjusting for age, body mass index (BMI), thyroid antibody positivity, and COH protocols. However, the preterm delivery rate was significantly higher in women with TSH < 2.5 mIU/L than in those with TSH ≥ 2.5 mIU/L, even after adjusting for relevant confounding factors. There was no significant difference in live birth weight between the two groups.

**Discussion:**

Mildly elevated TSH levels (TSH ≥ 2.5 mIU/L) after COH did not affect IVF/ICSI outcomes, and strict control of TSH levels within 2.5 mIU/L after COH might not be necessary. Additionally, strictly controlled TSH levels (TSH < 2.5 mIU/L) may increase preterm delivery risk.

## 1 Introduction

Adequate thyroid hormone levels are necessary for normal pregnancy and fetal development. Since the fetal thyroid gland is non-functional during early pregnancy, fetal growth and development are completely dependent on maternal thyroid hormone transfer. Thus, maternal hypothyroidism before and during pregnancy is associated with an increased risk of adverse pregnancy outcomes, including pregnancy loss, premature birth, and low birth weight (1, 2). Furthermore, intelligence quotient scores were reportedly lower in children of women with hypothyroidism than in children of women with normal thyrotropin concentrations, suggesting that hypothyroidism was detrimental to fetal neurocognitive development (3).

Given the potential danger of hypothyroidism, many studies suggest routine screening for thyroid function before and during pregnancy. In particular, serum thyroid-stimulating hormone (TSH) measurement is the most accurate assay for hypothyroidism evaluation because elevated levels of serum TSH are the earliest abnormal laboratory indicator of the occurrence of hypothyroidism (4). Although hypothyroidism is defined as an increase in serum TSH levels above the upper limit of normality, several guidelines suggest that the upper limit of TSH levels before pregnancy should not exceed 2.5 mIU/L; the Endocrine Society recommends that TSH should not exceed 2.5 mIU/L before pregnancy and during the first trimester (5). Furthermore, the 2011 American Thyroid Association guidelines recommend that the upper reference limit for serum TSH concentration in the first trimester of pregnancy should be defined as 2.5 mIU/L (6). The 2021 European Thyroid Association Guideline suggests levothyroxine (LT4) treatment in subfertile women with TSH levels >4.0 mIU/L to maintain serum TSH levels <2.5 mIU/L (7). Recently, large-scale cohort studies also indicated that mildly elevated TSH levels (TSH ≥2.5 mIU/L) before pregnancy may increase the risk of adverse pregnancy outcomes (8, 9). However, whether to monitor and control TSH levels during assisted reproductive treatment remains unclear.

An increasing number of studies reported a higher incidence rate of hypothyroidism in women with infertility, a population that needs to achieve pregnancy using assisted reproductive technology (ART) (10). Controlled ovarian hyperstimulation (COH) is a necessary procedure in ART, and whether thyroid function should be monitored during and after COH is controversial. The most important question is whether the changes in thyroid hormone levels during COH affect ART and pregnancy outcomes. In a meta-analysis of 15 cohort studies, Li et al. found significantly increased serum TSH levels and significantly decreased free thyroxine (FT4) levels during COH (11). Another meta-analysis of 14 cohort studies, which failed to detect differences in serum FT4 levels, also showed a significant increase in serum TSH levels in patients after COH (12). Although alteration in serum FT4 levels during COH remains debatable, a significant increase in serum TSH levels after COH has been observed in many studies.

Most published studies focused on the association between TSH levels before COH on ART outcomes. However, no large-scale prospective studies have evaluated the impact of TSH levels after COH on ART outcomes, which may be very important in fresh embryo transfer (ET) cycles. To address this clinical question, we performed this prospective study to assess whether TSH levels after COH affect ART outcomes in women undergoing fresh ET.

## 2 Methods

### 2.1 Study population

**Figure 1:**
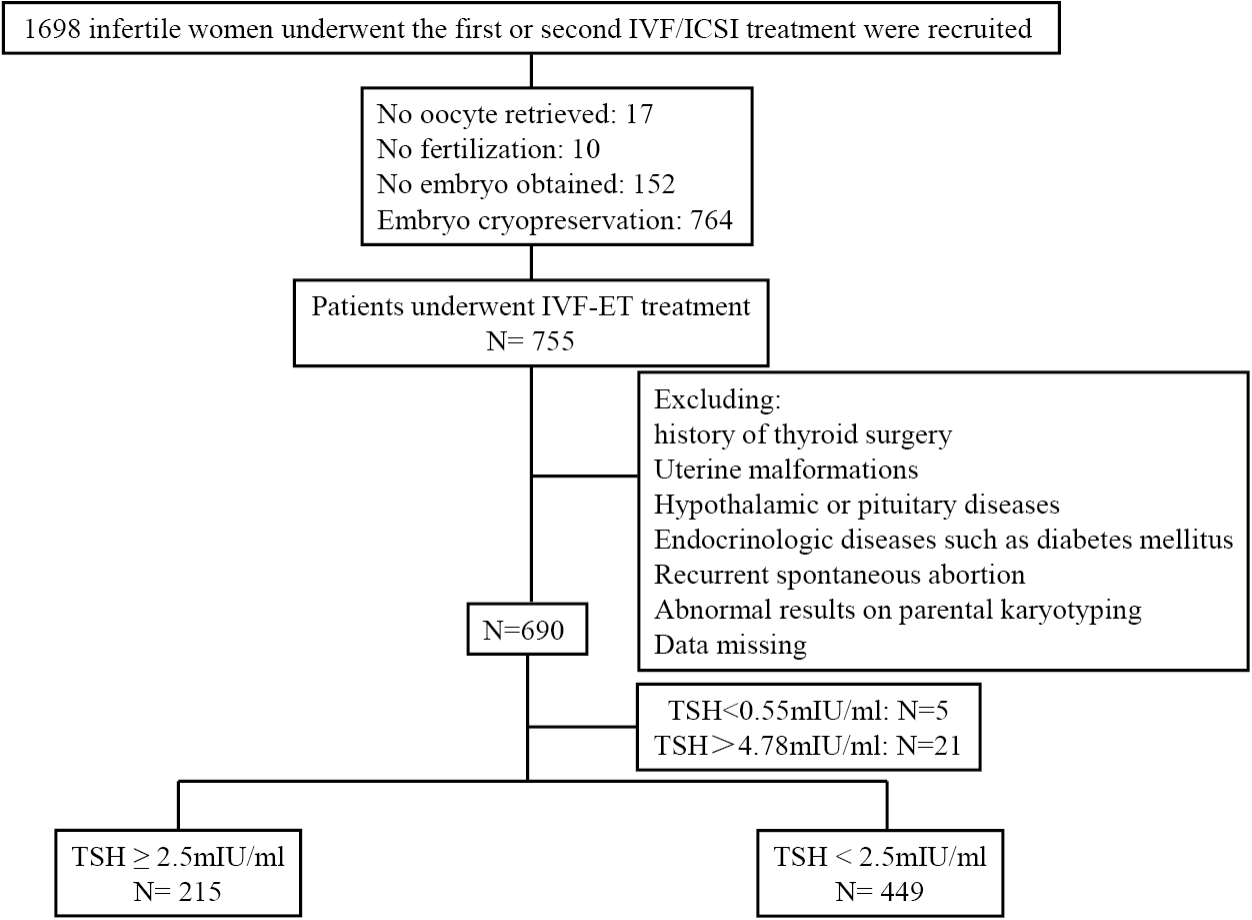
Flow chart of study cohort selection.

We recruited 1698 infertile women who underwent the first or second *in vitro* fertilization/intracytoplasmic sperm injection (IVF/ICSI) treatment and received fresh ET at Peking University Third Hospital between June and September 2021. Patients were eligible for this study if they were 20–40 years of age and scheduled for their first or second IVF/ICSI-ET cycle. Patients were excluded if they had a history of thyroid surgery, uterine malformations, hypothalamic or pituitary diseases, diabetes mellitus or other endocrinologic or metabolic diseases, recurrent spontaneous abortion (defined as three or more previous spontaneous pregnancy losses), and abnormal results on parental karyotyping. A total of 690 women were ultimately included (**Figure 1**). All patients were treated using standard COH protocols, including the ultralong gonadotropin-releasing hormone (GnRH) agonist protocol, long GnRH agonist protocol, or the GnRH antagonist protocol according to the patients’ conditions. Oocytes were retrieved 34–36 hours after administration of recombinant human chorionic gonadotropin (HCG). Insemination was performed 4–6 hours after oocyte retrieval by conventional IVF or ICSI, according to sperm quality. Up to two Day 3 embryos were transferred three days after oocyte retrieval.

### 2.2 Laboratory tests

Serum samples for thyroid hormone testing were collected after COH. Thyroid function measurements, including TSH, FT4, thyroid peroxidase antibody (TPOAb), and thyroglobulin antibody (TGAb) levels, were performed using a fully automatic chemiluminescence immunoassay analyzer (ADVIA Centaur XP, Siemens Healthcare Diagnostics). The reference range were 0.55–4.78 mIU/L for TSH and 0.89–1.80 ng/dL for FT4. The level below 60 IU/mL indicated negative for TPOAb and TGAb. Five patients with TSH <0.55 mIU/L and 21 patients with TSH >4.78 mIU/L were excluded. The patients were divided into a low-normal TSH group (TSH <2.5 mIU/L) and a high-normal TSH group (TSH ≥2.5 mIU/L).

### 2.3 Study outcomes

Clinical pregnancy was defined as the presence of at least one gestational sac in the uterus, identified using ultrasonography 35 days after ET. Miscarriage was defined as the loss of clinical pregnancy before 28 weeks of gestation. Live birth was defined as the delivery of at least one live fetus. Preterm delivery was defined as delivery before 37 weeks of gestation.

### 2.4 Statistical analyses

Continuous variables were expressed as means (standard deviations, SDs) for normally distributed data and as medians (interquartile ranges, IQRs) for data without a Gaussian distribution. Categorical variables were presented as numbers (percentages). Student’s t-test was used to compare the differences in continuous variables with normal distribution between the two groups, and the Mann-Whitney U test was performed for continuous variables without a Gaussian distribution. Comparisons between categorical variables were performed using the Chi-squared test. Logistic regression analysis was conducted to calculate the odds ratios (ORs) with 95% confidence intervals (CIs) after adjusting for relevant factors. A two-sided P<0.05 indicated statistical significance. All statistical analyses were performed using IBM SPSS Statistics for Windows version 24.0 software (Armonk, NY: IBM Corp).

## 3 Results

A total of 664 women who underwent IVF/ICSI treatment and fresh ET were included in this study. According to the TSH levels on HCG trigger day, 449 women with TSH <2.5 mIU/L were defined as the low-normal TSH group, and 215 women with TSH ≥2.5 mIU/L were defined as the high-normal TSH group. The baseline features of the two groups were compared, and the results were summarized in **Table 1**. Women with TSH ≥2.5 mIU/L had significantly higher body mass index (BMI, P=0.034) than women with TSH <2.5 mIU/L. No statistically significant differences were noted between the two groups in age, type of infertility, primary cause of infertility, rate of thyroid antibody positivity, or markers relevant to ovarian reserve, such as basal follicle-stimulating hormone, luteinizing hormone, estradiol, and anti-Mullerian hormone levels. We also collected data on thyroid function within six months before COH. While there was no significant difference in the basal FT4 level between two groups, a significantly higher basal TSH level was observed in the high-normal TSH group than in the low-normal TSH group (P<0.001).

**Table 1:** Baseline characteristics of patients.

Characteristics	Low-normal TSH (n = 449)	High-normal TSH (n = 215)	P value
Age, mean (SD), years	31.9 (3.7)	32.3 (3.8)	0.194
BMI, mean (SD), kg/m^2^	22.8 (3.5)	23.4 (3.6)	0.034^*^
Type of infertility, no. (%)			
Primary	281 (62.6)	130 (60.5)	0.599
Secondary	168 (37.4)	85 (39.5)	
Primary cause of infertility, no. (%) ^a^			
Male factors	166 (37.0)	83 (38.6)	0.448
Female factors			
Tubal factor	142 (31.6)	71 (33.0)	
Polycystic ovary syndrome	41 (9.1)	25 (11.6)	
Endometriosis	37 (8.2)	11 (5.1)	
Unknown factors	63 (14.0)	25 (11.6)	
Basal FSH, mean (IQR), mIU/mL ^b^	6.9 (5.7-8.2)	6.8 (5.6-8.5)	0.719
Basal LH, mean (IQR), mIU/mL	3.7 (2.5-5.0)	3.5 (2.3-5.0)	0.546
Basal estradiol, mean (IQR), pmol/L	146.5 (109.0-179.0)	141.0 (102.0-169.0)	0.136
Basal FT4, mean (SD), ng/dL ^c^	1.2 (0.2)	1.2 (0.2)	0.628
Basal TSH, mean (SD), mIU/L	1.8 (0.8)	2.8 (0.9)	< 0.001^*^
AMH, mean (IQR), ng/mL	2.1 (1.2-3.4)	1.8 (1.1-3.3)	0.284
No. of thyroid antibody positivity	64 (14.3)	28 (13.0)	0.668

AMH, anti-Mullerian hormone; BMI, body mass index; COH, controlled ovarian hyperstimulation; FSH, follicle-stimulating hormone; FT4, free thyroxine; IQR, interquartile range; LH, luteinizing hormone; no., number; SD, standard deviation; TSH, thyroid-stimulating hormone.

a Primary cause of infertility indicates the most important cause for patients seeking *in vitro* fertilization/intracytoplasmic sperm injection treatment.

b Testing for basal FSH, LH, and estradiol levels was performed between day 2 and day 4 of the menstrual cycle.

c Data of basal FT4 and TSH were collected within 6 months before COH.

^*^P<0.05.

The group characteristics of COH and IVF are presented in **Table 2**. We observed a significant difference in the terms of COH protocols between the two groups. The number of patients using GnRH antagonist protocols was significantly higher in the low-normal TSH group than in the high-normal TSH group. In contrast, the proportion of patients using ultralong GnRH agonists in the high-normal TSH group was higher than that in the low-normal TSH group. Although the gonadotropin dose and the days of ovarian stimulation were significantly higher in women with TSH ≥2.5 mIU/L than in those with TSH <2.5 mIU/L (P=0.007 and 0.041, respectively), no significant differences were observed between the two groups in the hormone levels on the HCG trigger day, the number of retrieved oocytes, and the number of good-quality embryos.

**Table 2:** Protocols of COH and data of IVF and ET.

Characteristics	Low-normal TSH (n = 449)	High-normal TSH (n = 215)	P value
Protocols of COH, no. (%)			
Ultralong GnRH agonist	25 (5.6)	24 (11.2)	0.035^*^
Long GnRH agonist	78 (17.4)	34 (15.8)	
GnRH antagonist	346 (77.1)	157 (73.0)	
Gonadotropin dose, median (IQR), IU	2625.0 (1875.0-3300.0)	2850.0 (2025.0-3900.0)	0.007^*^
No. of days of ovarian stimulation, median (IQR)	10.0 (9.0-12.0)	11.0 (9.0-13.0)	0.041^*^
LH level on HCG trigger day, median (IQR), mIU/mL	1.1 (0.5-2.3)	1.2 (0.5-2.4)	0.443
Estradiol level on HCG trigger day, median (IQR), pmol/L	6099.0 (4649.0-8449.0)	6637.0 (4883.0-8427.0)	0.186
Progesterone level on HCG trigger day, median (IQR), nmol/L	1.5 (1.3-1.9)	1.6 (1.3-1.9)	0.721
No. of retrieved oocytes per cycle, median (IQR)	9.0 (6.0-12.0)	9.0 (6.0-11.0)	0.632
No. of good-quality embryos, median (IQR)^a^	4.0 (2.0-5.0)	3.0 (2.0-5.0)	0.383
No. of embryo transferred, no. (%)			
1	57 (12.8)	21 (9.9)	0.278
2	389 (87.2)	192 (90.1)	

COH, controlled ovarian hyperstimulation; IVF, *in vitro* fertilization; ET, embryo transfer; GnRH, gonadotropin-releasing hormone; HCG, human chorionic gonadotropin; IQR, interquartile range; LH, luteinizing hormone; no., number; SD, standard deviation; TSH, thyroid-stimulating hormone.

a The embryos were evaluated on the third day after fertilization. Good-quality embryos were developed from two pronuclei zygotes and met the following criteria: (1) had more than five blastomeres; (2) size difference <20%; and (3) fragmentation <50%.

^*^P<0.05.

There were no significant differences between the low-normal TSH and high-normal TSH groups in the rates of clinical pregnancy (50.1% vs. 44.2%, P=0.153), miscarriage (16.4% vs. 14.7%, P=0.703), and live birth (41.9% vs. 37.7%, P=0.303) (**Table 3**). Multiple logistic regression was performed to adjust for relevant confounding factors, and the results showed no significant differences between the two groups in the rates of clinical pregnancy (OR: 0.80, 95% CI: 0.58–1.12, P=0.193), miscarriage (OR: 0.84, 95% CI: 0.42–1.68; P=0.619), and live birth (OR: 0.87, 95% CI: 0.62–1.23, P=0.434) after adjusting for age, BMI, thyroid antibody positivity, and COH protocols (**Table 4**). Surprisingly, a significantly higher rate of preterm delivery was observed in women with TSH <2.5 mIU/L compared with those with TSH ≥2.5 mIU/L (22.3% vs. 11.1%, P=0.031), and this difference existed after adjusting for age, BMI, thyroid antibody positivity and COH protocols (OR: 0.39, 95% CI: 0.18–0.86, P=0.020) (**Tables 3**, **4**). In singleton or twin pregnancies, birth weight was not significantly different between the two groups.

**Table 3:** Pregnancy outcomes of women with low-normal TSH levels and those with high-normal TSH levels.

Outcomes	Low-normal TSH (n=449)	High-normal TSH (n=215)	P value
Clinical pregnancy ^a^, no. (%)	225/449 (50.1)	95/215 (44.2)	0.153
Miscarriage ^b^, no. (%)	37/225 (16.4)	14/95 (14.7)	0.703
Live birth ^c^, no. (%)	188/449 (41.9)	81/215 (37.7)	0.303
Preterm delivery ^d^, no. (%)	42/188 (22.3)	9/81 (11.1)	0.031^*^
Birth weight, g			
Singleton	3300.0 (3000.0-3600.0)	3350.0 (2990.0-3600.0)	0.908
Twin	2550.0 (2300.0-2745.0)	2525.0 (2150.0-2703.8)	0.365

No., number; TSH, thyroid-stimulating hormone.

a Clinical pregnancy was defined as the presence of at least one gestational sac in the uterus identified using ultrasonography 35 days after embryo transfer.

b Miscarriage was defined as the loss of a clinical pregnancy before 28 weeks of gestation. The miscarriage rate was defined as the proportion of women with miscarriage among women with clinical pregnancy.

c Live birth was defined as the delivery of at least one living fetus.

d Preterm delivery was defined as delivery before 37 weeks. The preterm delivery rate was defined as the proportion of women with preterm delivery among women with live birth.

^*^P < 0.05.

**Table 4:** Multivariate logistic regression analysis of factors associated with pregnancy outcomes.

Factors	Clinical pregnancy		Miscarriage		Live birth		Preterm delivery	
	OR (95% CI)	P value	OR (95% CI)	P value	OR (95% CI)	P value	OR (95% CI)	P value
Age, years	0.95 (0.91-0.99)	0.011^*^	1.11 (1.01-1.21)	0.023^*^	0.93 (0.89-0.97)	0.001^*^	0.97 (0.88-1.06)	0.435
BMI, kg/m^2^	0.98 (0.94-1.03)	0.455	1.07 (0.98-1.16)	0.160	0.97 (0.93-1.02)	0.200	1.07 (0.98-1.17)	0.146
TSH on HCG trigger day								
Low-normal (reference)	NA	NA	NA	NA	NA	NA	NA	NA
High-normal	0.80 (0.58-1.12)	0.193	0.84 (0.42-1.68)	0.619	0.87 (0.62-1.23)	0.434	0.39 (0.18-0.86)	0.020^*^
Thyroid antibody positivity	1.00 (0.64-1.56)	0.998	0.31 (0.09-1.05)	0.060	1.25 (0.80-1.96)	0.332	1.54 (0.68-3.46)	0.300
Protocols								
Ultralong GnRH agonist	1.30 (0.72-2.36)	0.383	1.17 (0.40-3.42)	0.774	1.17 (0.64-2.15)	0.613	1.44 (0.43-4.77)	0.554
Long GnRH agonist	1.27 (0.84-1.92)	0.252	0.99 (0.44-2.21)	0.974	1.25 (0.82-1.90)	0.296	1.09 (0.49-2.41)	0.834
GnRH antagonist	NA	NA	NA	NA	NA	NA	NA	NA

BMI, body mass index; CI, confidence interval; GnRH, gonadotropin-releasing hormone; HCG, human chorionic gonadotropin; NA, not available; OR, odds ratio; TSH, thyroid-stimulating hormone.

^*^P<0.05.

## 4 Discussion

Currently, whether screening for thyroid function after COH is needed to initiate appropriate management and intervention remains controversial. To our knowledge, this is the first large-scale prospective study to assess the impact of maternal TSH levels after COH on IVF/ICSI outcomes in women undergoing fresh ET. In this cohort of women undergoing IVF/ICSI-ET treatment, women with TSH ≥2.5 mIU/L after COH had similar pregnancy outcomes to those of women with TSH <2.5 mIU/L. Unexpectedly, women with TSH <2.5 mIU/L had a higher preterm delivery risk, even after adjusting for relevant confounders, such as age, BMI, thyroid antibody positivity, and COH protocols. Our study suggests that TSH ≥2.5 mIU/L after COH is not a risk factor for poorer reproductive outcomes, and there is no need to strictly control TSH levels below 2.5 mIU/L after COH.

The impact of mildly elevated TSH levels on pregnancy outcomes has been debated for several years; most studies have focused on the impact of preconception TSH values on pregnancy outcomes. Two large-scale population-based cohort studies classified women into different groups according to TSH levels within 6 months before pregnancy and showed that even slightly elevated preconception TSH levels were associated with various adverse maternal outcomes, and recommended an optimal preconception TSH range between the lower reference limit and 2.50 mIU/L (8, 9). However, no large-scale study has investigated the effect of TSH levels after COH treatment on pregnancy outcomes.

COH is an important part of ART, which causes estradiol to rapidly rise to supraphysiological levels. Many studies have shown an association between COH and changes in thyroid function (11, 12). The underlying mechanism involves an increase in thyroid-binding globulin levels triggered by supraphysiologic estradiol levels, which reduces free thyroid hormone concentrations and, in turn, triggers serum TSH elevation. Unlike previous studies, our study analyzed TSH levels after COH instead of before, which may better reflect thyroid function before fresh ET. We did not find statistically significant differences in the rates of clinical pregnancy, miscarriage, or live birth between the low-normal and high-normal TSH groups. Our study agrees with several previous studies that showed an association between TSH levels before COH and IVF/ICSI outcomes in infertile women. Based on a secondary data analysis of 1468 infertile women, Seungdamrong et al. showed that preconception TSH ≥2.5 mIU/L did not affect conception, clinical pregnancy, miscarriage, and live birth rates in infertile women without reproductive treatment (13). Two other studies, which restricted the patient populations to infertile women undergoing IVF/ICSI treatment, also found no significant differences in the rates of clinical pregnancy, miscarriage, and live birth between the TSH <2.5 mIU/L and TSH ≥2.5 mIU/L groups (14, 15).

Most previous studies focused on the impact of the mildly elevated TSH levels on adverse ART outcomes and some guidelines recommended to maintain TSH levels below 2.5 mIU/L before pregnancy. In our study, no significant difference was found in the rates of clinical pregnancy, miscarriage and live birth between women with TSH ≥2.5 mIU/L and women with TSH <2.5 mIU/L, however, we observed a significantly higher rate of preterm delivery in women with TSH <2.5 mIU/L than in those with TSH ≥2.5 mIU/L, even after adjusting for age, BMI, thyroid antibody positivity, and COH protocols, which suggest that strictly control TSH levels below 2.5 mIU/L may trigger a detrimental effect on assisted reproductive outcomes. Our results were supported by a recent large-scale cohort study, which enrolled 175,112 women and performed a restricted cubic spline (RCS) regression model with multiple percentiles of TSH level to analyze the association between preconception TSH levels and adverse pregnancy outcomes. The study showed lower TSH was associated with a higher OR of preterm delivery (nonlinear P<0.001) (16). However, some previous studies based on TSH levels before COH showed inconsistent results. In a retrospective study, Zhang et al. reported no significant difference in the rates of preterm delivery between women with low TSH levels (TSH <2.5 mIU/L) and those with mildly elevated TSH levels (TSH ≥2.5 mIU/L) (14). Another retrospective study showed a significantly lower gestational age at delivery and lower birth weight in women with TSH ≥2.5 mIU/L than in those with TSH <2.5 mIU/L before COH (17). The controversial conclusions may result from the different timing of TSH test and screening for TSH levels after COH may be necessary in fresh ET cycles.

In our study, mildly elevated TSH levels (TSH ≥2.5 mIU/L) after COH did not affect IVF/ICSI outcomes. However, the rate of preterm delivery significantly increased in women with TSH <2.5 mIU/L. Based on our study, TSH levels after COH should not be strictly reduced to below 2.5 mIU/L. All assays for TSH and antithyroid antibodies in our study were performed in the same standardized laboratory, which reduced the potential inter-assay variability. However, our study also had some limitations. First, we only focused on TSH levels after COH and did not collect longitudinal TSH measurements throughout pregnancy. However, many previous studies have already shown the impact of TSH levels during different stages of pregnancy on reproductive outcomes (18). Second, we did not investigate the impact of the use of levothyroxine because of marked differences in the initiation time of thyroid hormone replacement. As such, further randomized controlled trials may be needed. Furthermore, we did not analyze additional pregnancy complications, such as gestational hypertension and gestational diabetes mellitus, because of the relatively small sample size. Further prospective studies with larger sample sizes are required to confirm these findings.

## 5 Conclusions

To our knowledge, our study is the first large-scale prospective study to demonstrate that mildly elevated TSH levels after COH may not adversely affect IVF/ICSI outcomes in women who receive fresh ET, that TSH ≥2.5 mIU/L after COH is not a risk factor for poorer reproductive outcomes, and that strict control of TSH levels to levels below 2.5 mIU/L after COH may not be necessary.

## Data availability statement

The original contributions presented in the study are included in the article/supplementary material, further inquiries can be directed to the corresponding authors.

## Ethics statement

The studies involving humans were approved by Peking University Third Hospital Medical Science Research Ethics Committee. The studies were conducted in accordance with the local legislation and institutional requirements. The participants provided their written informed consent to participate in this study. Written informed consent was obtained from the individual(s) for the publication of any potentially identifiable images or data included in this article.

## Author contributions

NH took part in patient follow-up and wrote the initial draft of the paper. NH, LC and YL contributed to the data analysis. HC and JQ contributed to the conception and design of the study. All authors contributed to the research discussion and manuscript revision.
